# Hydration Behavior of Magnesium Potassium Phosphate Cement: Experimental Study and Thermodynamic Modeling

**DOI:** 10.3390/ma15238496

**Published:** 2022-11-29

**Authors:** Jinrui Zhang, Wenjun Niu, Zhen Liu, Youzhi Yang, Wujian Long, Yuanyuan Zhang, Biqin Dong

**Affiliations:** 1 State Key Laboratory of Hydraulic Engineering Simulation and Safety, Tianjin University, Tianjin 300072, China; 2 Guangdong Province Key Laboratory of Durability for Marine Civil Engineering, Shenzhen University, Shenzhen 518060, China

**Keywords:** magnesium potassium phosphate cement, hydration, material characterization methods, thermodynamic modeling, K-struvite

## Abstract

The microstructure and performance of magnesium potassium phosphate cement (MKPC), a kind of magnesium phosphate cement (MPC), are determined by the hydration products. In this paper, the hydration behavior of MKPC is investigated through various material characterization methods and thermodynamic modeling, including X-ray diffraction (XRD), thermogravimetric and differential scanning calorimeter (TG/DSC), scanning electron microscopy (SEM), mercury intrusion porosimetry (MIP) and GEMS software. The results of XRD, TG/DSC and SEM all indicate that K-struvite (MgKPO_4_·6H_2_O) is the main hydration product of MKPC. When the curing age is 1 day and 28 days, the TG data indicate that the mass loss of MKPC in the range of 60–200 °C is 17.76% and 17.82%, respectively. The MIP results show that the porosity of MKPC is 29.63% and 29.61% at the curing age of 1 day and 28 days, respectively, which indicates that the structure of MKPC becomes denser with the increase in curing age. In addition, the cumulative pore volume of MKPC at the curing age of 28 days is 2.8% lower than that at 1 day, and the pore diameters are shifted toward the small pores. Furthermore, the thermodynamic modeling is well suited to make an analysis of the hydration behavior of MKPC.

## 1. Introduction

Magnesium phosphate cement (MPC) is a new type of cement-based material that is formed by hardening in the presence of water through the reaction between dead burnt magnesium oxide (MgO) and phosphate, which is also called chemically bonded phosphate ceramic/cement [1,2,3]. Compared with ordinary Portland cement, MPC has numerous outstanding properties, e.g. high strength at an early age [4,5,6], rapid setting [7], strong bonding to concrete substrates [8,9,10], and excellent durability [11]. Therefore, it is widely used in various fields, including the repair of civil structures [7], the stabilization/solidification (S/S) of low-level nuclear wastes and heavy metal wastes [12,13,14], treatment of waste water [15] and restorations of teeth and bones [16,17,18]. Generally, the representative phosphates are ammonium dihydrogen phosphate (NH_4_H_2_PO_4_) and potassium dihydrogen phosphate (KH_2_PO_4_) [19]. However, NH_4_H_2_PO_4_ generates unpleasant gases during its use, which harm the environment and seriously hinder its application in buildings [20,21,22]. In contrast, the environmental pollution problem can be well avoided by KH_2_PO_4_, and the use of KH_2_PO_4_ can result in MPC with higher early strength and relatively slower reaction rates [23,24,25]. In addition, with the benefit of a smaller dissociation constant and a lower solubility of KH_2_PO_4_, the reaction of KH_2_PO_4_ with dead burnt MgO is easier to control [26]. The cement produced by the reaction of KH_2_PO_4_ with dead burnt MgO is magnesium potassium phosphate cement (MKPC). The main reaction process of MKPC is given by Equation (1) [27].

MgO + KH_2_PO_4_ + 5H_2_O → MgKPO_4_·6H_2_O (1)

As shown in Equation (1), MgKPO_4_·6H_2_O (K-struvite) is the main product of hydration of MKPC and provides the main strength support [28]. Studies have shown that the generation of K-struvite is accompanied by the formation of other intermediate products, e.g. MgHPO_4_·7H_2_O and Mg_2_KH(PO_4_)·15H_2_O [29]. However, for the convenience of the study, Equation (1) can still be taken as the only process of hydration of MKPC [20]. In recent years, the excellent properties of MKPC have attracted more attention to its hydration behavior. At the same time, various material characterization methods have been applied to study the hydration process of MKPC, e.g. X-ray diffraction (XRD), thermogravimetric and differential scanning calorimeter (TG/DSC), scanning electron microscopy (SEM), mercury intrusion porosimetry (MIP), and so on [30]. The results obtained by experimental methods not only reveal the hydration behavior of MKPC, but also can be combined with numerical methods, e.g. thermodynamic modeling.

Thermodynamic models have been widely used for ordinary Portland cement, calcium sulfoaluminate cements, and alkali activated systems, and these thermodynamic models have effectively promoted the study of cement hydration [31,32,33,34]. Thermodynamic equilibrium calculations can realize the prediction of the composition of solid, gas, and liquid phases in the system at specific temperatures and pressure levels. Based on this, the hydration of MKPC, mainly reaction (1), is simulated numerically in this paper.

Two basic equations for solving geochemical equilibria are relied on by existing thermodynamic modeling software [34]:

(1) Law of mass action (LMA) equation:

(2) $$v=kc_{A}^{a}c_{B}^{b}$$

where *v* is the reaction rate and *k* is the reaction rate constant. For a primitive reaction: *aA* + *bB* → products, *c_A_* and *c_B_* are the concentrations of materials *A* and *B*, respectively.

The LMA equation demonstrates that the reaction rate of a simple chemical reaction is influenced by the mass concentration of the reactants, which is proportional to the product of the mass concentrations of the reactants. This method is mostly used in geochemical codes, e.g. PHREEQC and EQ3/6 [35,36,37].

(2) Minimization of Gibbs free energy of the system:

(3) ΔG=ΔH−TΔS

where ΔG is the Gibbs free energy change, ΔH is the enthalpy change, *T* is the temperature and ΔS is the entropy change.

Under isothermal-isobaric conditions, and without any other work, the system always proceeds spontaneously in the direction of decreasing Gibbs energy until it reaches equilibrium. This thermodynamic modeling approach is mostly used in the thermodynamic calculations of GEMS [38,39,40].

In summary, this paper investigates the hydration process of MKPC through a series of material characterization methods and thermodynamic modeling approaches. Considering that the Gibbs free energy minimization method does not require pre-assumptions on internal phases, solid solvents, pH or redox potential of the system, and its results also follow the same conservation of the mass and charge of the system, the thermodynamic modeling of the hydration process is carried out using GEMS software (v.3) in this paper.

## 2. Materials and Methods

### 2.1. Materials

The MgO used in this paper was produced by Jinan Ludong Refractory Co., Ltd. (Shandong, China) and calcined from magnesite, whose main component is MgCO_3_. It is worth noting that the MgO obtained by this method would react rapidly with KH_2_PO_4_ when used directly without treatment, making it difficult to form an ideal MKPC. It was found that calcination of MgO at a higher temperature could improve the reactivity of MgO, thus allowing simultaneous hydration with other cementing components and reducing internal defects [22]. Therefore, the MgO used in the preparation of MKPC in this paper was calcined at 1500–1600 °C. The chemical composition was determined by X-ray fluorescence (XRF), and the results are shown in Table 1. Analytical-grade KH_2_PO_4_, a white crystalline powder with 99% purity, was obtained from Sinopharm Group Chemical Reagent Co., Ltd. (Shanghai, China). The water was laboratory water.

When retarders are not considered, low M/P ratios result in the presence of KH_2_PO_4_ in MKPC, which has a negative impact on the material, while high M/P ratios provide very satisfactory results, resulting in good mechanical properties and dimensional stability of MKPC [41]. In this paper, MKPC was prepared considering that the M/P molar ratio is 8.5, and the water-to-cement ratio is 0.22. In order to ensure the homogeneity of MKPC, MgO and KH_2_PO_4_ were mixed in advance and stirred well. First, they were dry mixed for 2 min, then water was added and slow stirred for 1 min, followed by 30 s of fast stirring to prepare MKPC that met the requirements. The prepared MKPC was poured into the pre-prepared plastic molds and covered with cling film after vibrating homogeneously. The plastic molds were removed after 24 h, and the specimens were cured at room temperature for different amounts of time.

### 2.2. Methods

#### 2.2.1. X-ray Diffraction (XRD)

XRD can be used to determine the crystalline phase in MKPC. Different levels of diffraction are produced when different substances are irradiated with X-rays, and different hydration products can be distinguished according to their diffraction patterns. Samples of different curing ages were ground and preserved in a sealed bottle by soaking in anhydrous ethanol, which was used to prevent the continuation of the hydration. In this paper, XRD analysis of the dried solid phase was performed by Cu-Kα radiation (wavelength of 1.541874 Å) by using an X’Pert PRO MPD diffractometer (Nalytical, The Netherlands). The stepping size used in this paper was 0.02° in the 2θ range, ranging from 10° to 90°.

#### 2.2.2. Thermogravimetric and Differential Scanning Calorimetry (TG/DSC)

Under a program-controlled temperature, TG analysis is a kind of thermal analysis technique that can measure the change in mass of a sample versus temperature, which is used to study the thermal stability and composition of a material. DSC is a technique that uses a program to measure the power difference between a substance and a reference material as a function of temperature, at a controlled temperature. In this paper, the NETZSCH STA449C instrument (Netzsch-Gerätebau GmbH, Selb, Germany) was used to measure the heating of dried sample powders of different curing ages in the range of 30–1000 °C, with a heating rate of 10 °C/min.

#### 2.2.3. Scanning Electron Microscopy (SEM)

SEM is imaged by modulation of various physical signals excited by a finely focused electron beam as it scans over the surface of the sample. With its high magnification, it can directly observe the fine structure of the sample and, thus, better observe the morphology of different hydration products. In this paper, samples taken from crushed MKPC at different curing ages were observed separately on a Zeiss Gemini 300 instrument (Carl Zeiss, Oberkochen, Germany), in order to investigate their morphology and elemental composition. Furthermore, the evaporated gold was covered on the surface of the samples to enhance the electrical conductivity and obtain a better observation.

#### 2.2.4. Mercury Intrusion Porosimetry (MIP)

Mercury, which is not infiltrated into general solids, can enter the hole by applying external pressure. The larger the external pressure, the smaller the diameter of the hole into which mercury can enter. The volume of the pore is judged by measuring the amount of mercury entering the pore under different external forces. In this paper, mercury pressure experiments were performed on MIP samples in order to evaluate the pore structure of MKPC by the AutoPore 9500 (Micromeritics, Norcross, GA, USA).

#### 2.2.5. Thermodynamic Modeling

Thermodynamic modeling was performed using the Gibbs free energy minimization software (GEMS, v.3) for the purpose of predicting the composition of matter within the MKPC at equilibrium. Lothenbach et al. summarized the Cemdate18 database, which is applicable to hydrated Portland, calcium aluminate, calcium sulfoaluminate, blended cements and alkali activated materials, and demonstrated that an accurate and complete thermodynamic database effectively aided thermodynamic modeling in completing the prediction of the substance and the chemical composition of the cement after hydration [42]. Therefore, for reaction (1), the accuracy and completeness of the thermodynamic data of the phases in the system are important factors affecting the thermodynamic calculations. In addition, the thermodynamic database of magnesium phosphate hydrate was developed by Lothenbach et al., and greatly facilitated the study of MKPC [43]. Thermodynamic data for several substances of interest, including magnesium hydroxide (Mg(OH)_2_), MgO, KH_2_PO_4_ and K-struvite, are shown in Table 2. These data have potential for use in this paper.

## 3. Results and Discussion

### 3.1. Reaction Products in Hardened MKPC

**Figure 1:**
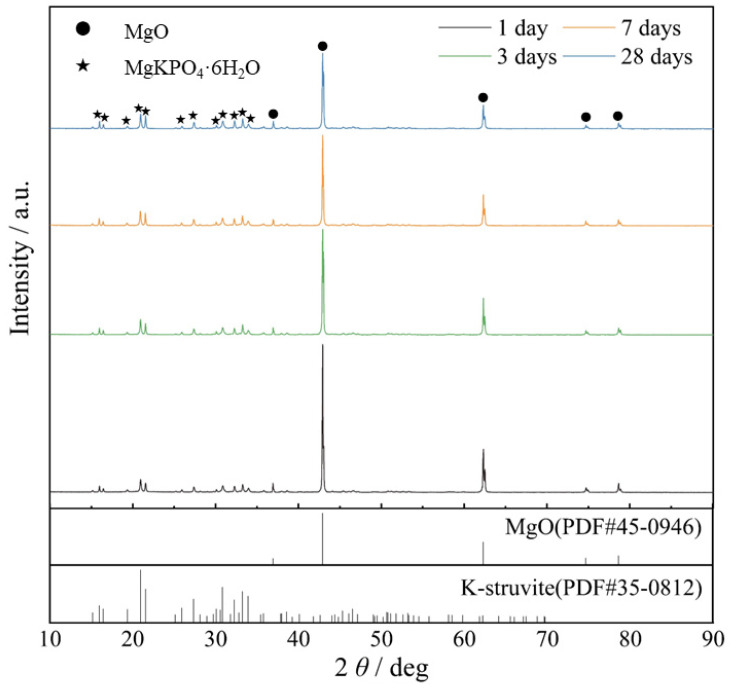
XRD patterns of MKPC at different curing ages.

Figure 1 shows the XRD patterns of MKPC at different curing ages. As shown in Figure 1, for MKPC, K-struvite is the main hydration crystallization product, which is the same as the results of previous studies [44,45,46]. The XRD patterns corresponding to MgO are clearly observed in each plot at different curing ages, indicating that a large amount of unreacted MgO still exists in the system at different times. With the increase in the curing age, the peak diffraction intensity corresponding to MgO decreases, and that corresponding to K-struvite increases. This indicates that the reaction is still continuing, but the rate of reaction is gradually decreasing. In fact, XRD measures the crystalline phase within the MKPC, while some studies have indicated that the hydration product K-struvite exists in both crystalline and amorphous forms in the MKPC, and both together contribute to the strength development of MKPC [47]. However, as a whole, the intensity of the characteristic peaks of K-struvite crystals in Figure 1 is weak, without high intensity sharp peaks, which is a result of the poor crystallinity of the hydration product caused by the relatively high M/P molar ratio of the MKPC in this study [48].

**Figure 2:**
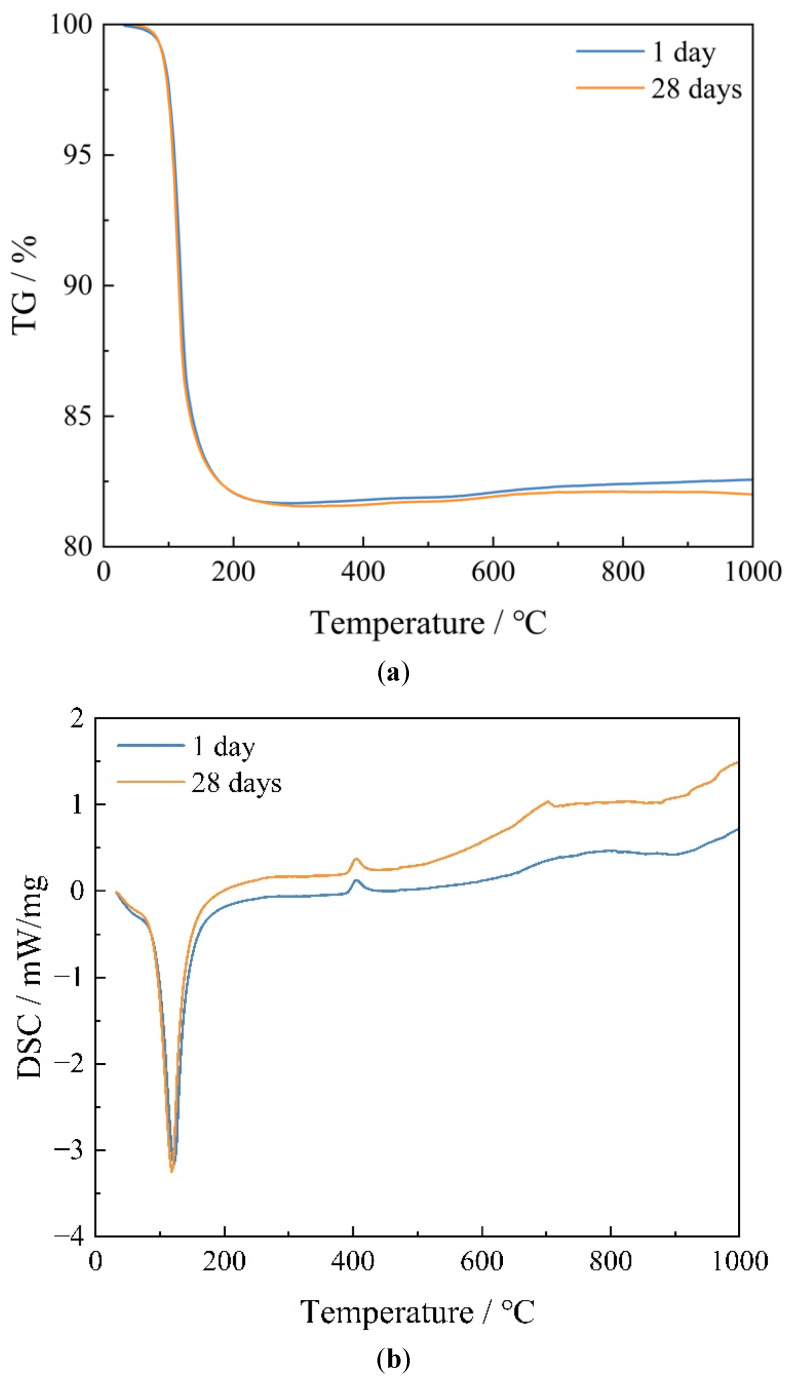
TG/DSC curves of MKPC at different curing ages. (**a**) TG curves; (**b**) DSC curves.

Figure 2 shows the results of TG/DSC for MKPC. It can be seen that the TG/DSC curves of MKPC at different curing ages are closely similar, with significant mass loss occurring below 200 °C. Among them, the mass loss of MKPC in the range of 60–200 °C is 17.76% and 17.82% at the curing age of 1 day and 28 days, respectively. In addition, the DSC curves show that there is a significant heat absorption peak near 120 °C. This is all a result of the dehydration of the hydration product K-struvite [49]. The decomposition is as follows:

MgKPO_4_·6H_2_O → MgKPO_4_ + 6H_2_O↑ (4)

The DSC results reveal that there are small exothermic peaks at about 400 and 700 °C, but the TG results show no mass change. This is because K-struvite transforms to magnesium phosphate (Mg_3_(PO_4_)_2_) and magnesium pyrophosphate (Mg_2_P_2_O_7_) under high temperature conditions after losing the crystal water with the increase in temperature. It transforms to Mg_3_(PO_4_)_2_ at about 400 °C and to Mg_2_P_2_O_7_ at about 700 °C. The TG curves corresponding to MKPC at different curing ages basically overlap, as do the heat absorption peaks in the DSC curves, which are only slightly different. This indicates that the hydration reaction of MKPC mainly occurs before 1 day, and that the rate of the hydration reaction after 1 day is low, which is consistent with the XRD results.

### 3.2. Microstructure Analysis of MKPC

**Figure 3:**
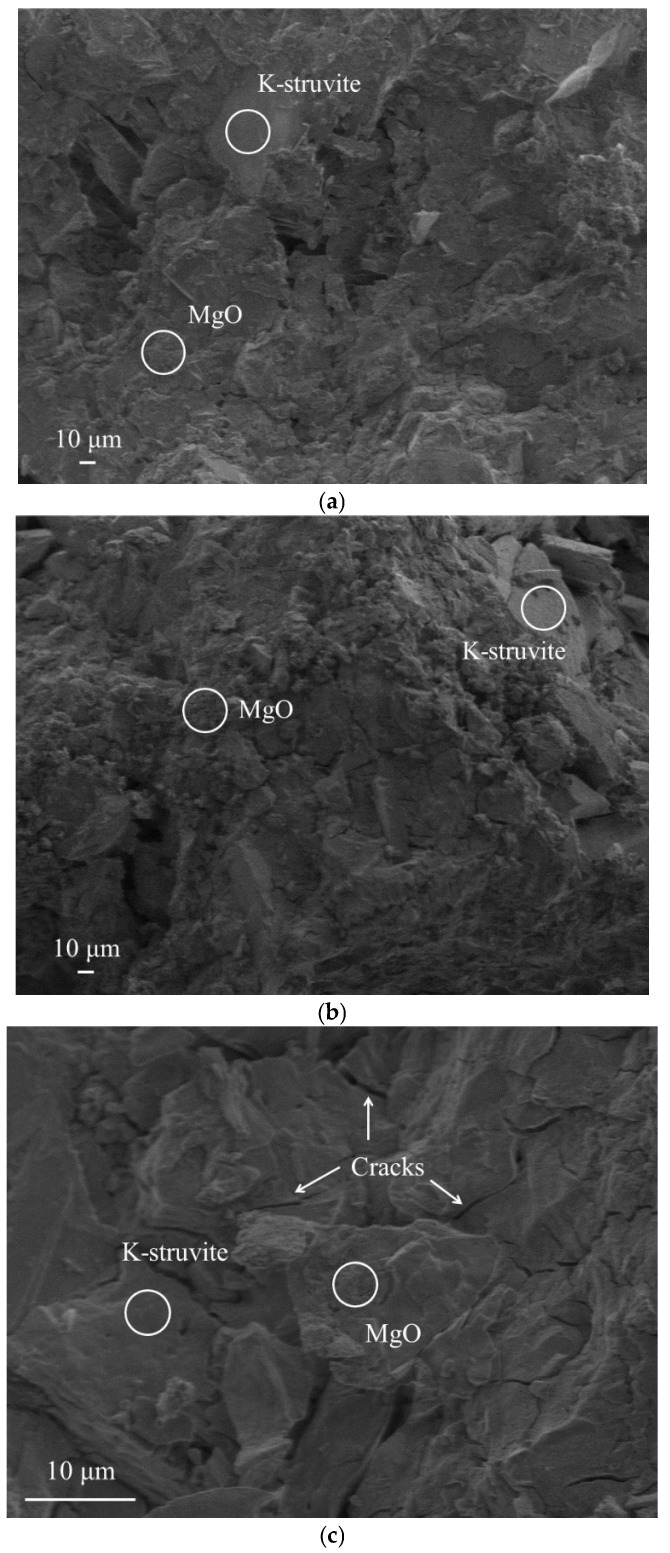
The microstructure of MKPC. (**a**) 1 day, ×300; (**b**) 28 days, ×300; (**c**) 28 days, ×2000.

Figure 3 shows the microstructure of MKPC obtained by SEM. It cannot be ignored that both the hydration product K-struvite and the unreacted raw material MgO are clearly observed in the SEM images, where the unreacted MgO appears granular and the hydration product K-struvite appears clearly plate-like [20]. The surplus of MgO is a result of the high M/P molar ratio in this study with the addition of excess MgO, which causes the KH_2_PO_4_ in the system to react completely, thus improving the mechanical strength of MKPC [50]. Figure 3a,b show that the structure of MKPC is slightly denser with the increase in the curing age. The presence of cracks can be seen in Figure 3c, which are considered by Ma et al. to be gaps left between adjacent particles during the partial connection of the crystalline K-struvite [51].

### 3.3. Pore Structure Analysis of MKPC

**Figure 4:**
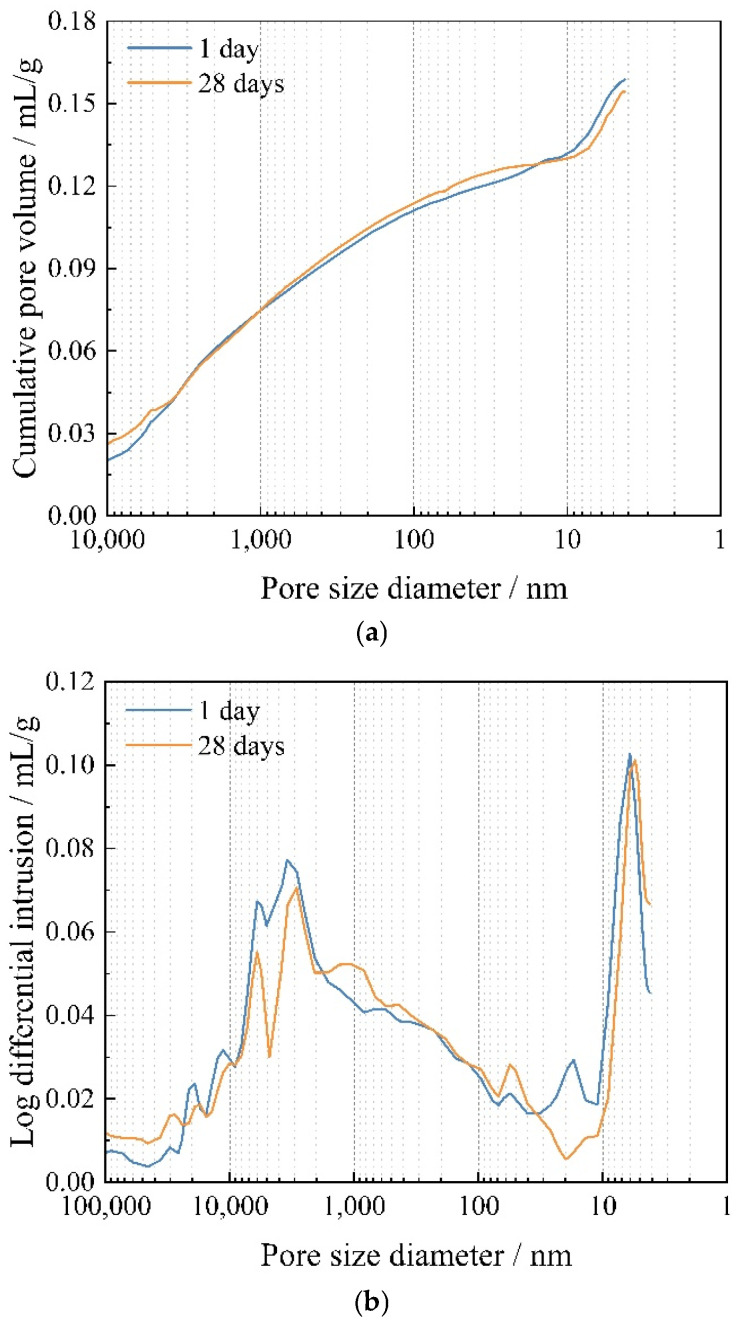
The pore structure of MKPC. (**a**) Cumulative pore volume distribution of the MKPC; (**b**) log differential intrusion of the MKPC.

The MIP results show that the porosity of MKPC is 29.63% and 29.61% at the curing age of 1 day and 28 days, respectively. Figure 4 shows the cumulative pore volume curves and pore size distribution curves of MKPC at the curing age of 1 day and 28 days. It can be seen that the cumulative pore volume of the MKPC decreases with the increase in the curing age. The pore size distribution curves can be used for qualitative comparison, and Figure 4b shows that the pore size distribution shifts toward the direction of small pores [52]. It indicates that the structure of MKPC becomes denser with increasing hydration time, which is caused by the continuous reaction of the constituents of MKPC with water during this period. However, it is worth mentioning that the cumulative pore volume at the curing age of 28 days is only 2.8% lower than that at the curing age of 1 day, which also suggests that the hydration reaction rate of MKPC after the curing age of 1 day is slow, and its main reaction process has occurred before 1 day. The cumulative pore volume curves grow relatively uniformly in the interval of 10,000–10 nm, while the growth becomes suddenly larger in the range of less than 10 nm, which demonstrates that there are more small diameter pores distributed in the MKPC.

### 3.4. Thermodynamic Modeling Analysis

**Figure 5:**
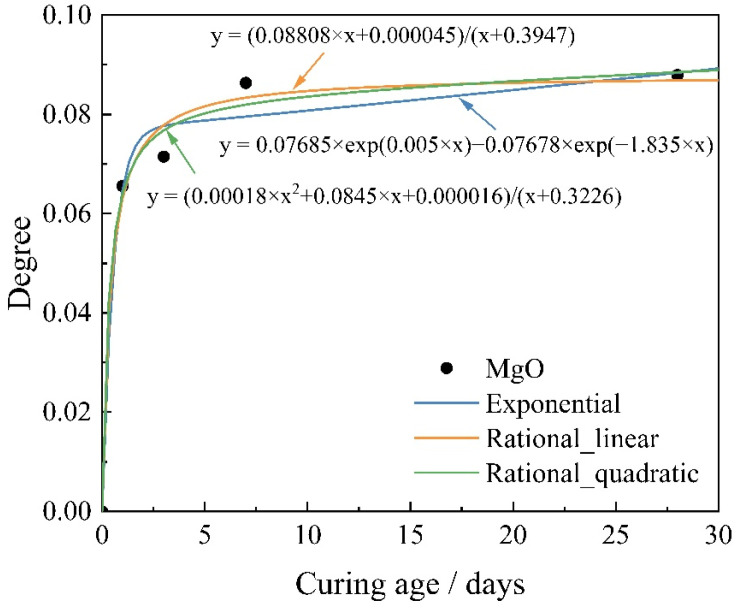
The reaction degree of MgO fitted by different form functions.

Thermodynamic calculations of GEMS require the variation of the reactant content as a function of time to be entered into the software. In this paper, the degree of reactivity of reactant MgO is calculated by analyzing the content of MgO in MKPC at different curing ages based on XRD and TG/DSC data. After that, the reaction degree of MgO is fitted by different forms of functions, which are exponential functions, linear rational number approximation and quadratic rational number approximation, respectively. The forms of the different functions are given by Equations (5)–(7). Figure 5 shows the different forms of function fitting for the degree of MgO reaction.

(1) Exponential fitting:

(5) y=a×exp(b×x)+c×exp(d×x)

where *a*, *b*, *c* and *d* are all fitting coefficients, *x* is the curing age and *y* is the reaction degree of MgO.

(2) Linear rational number approximation:

(6) $$y=(p_{1}\times x+p_{2})/(x+q_{1})$$

where *p*_1_, *p*_2_ and *q*_1_ are all fitting coefficients, *x* is the curing age and *y* is the reaction degree of MgO.

(3) Quadratic rational number approximation:

(7) y=(p1×x^2+p2×x+p3)/(x+q1)

where *p*_1_, *p*_2_, *p*_3_ and *q*_1_ are all fitting coefficients, *x* is the curing age and *y* is the reaction degree of MgO.

In order to better select the appropriate function to represent the reaction degree of MgO, the sum of squared errors (*SSE*), coefficient of determination (*R*^2^), adjusted coefficient of determination (*Adjusted R*^2^), and root mean squared error (*RMSE*) are compared under different forms of function fitting, and their expressions are as follows:

(1) Sum of squared errors (*SSE*):

(8) $$SSE={\sum_{i=1}^{n}\left({\hat{y}}_{i}-y_{i}\right)}^{2}$$

(2) Coefficient of determination (*R*^2^):

(9) $$R^{2}=1-\frac{RSS}{TSS}=1-\frac{\sum_{i=1}^{n}{\left({\hat{y}}_{i}-y_{i}\right)}^{2}}{\sum_{i=1}^{n}{\left({\bar{y}}_{i}-y_{i}\right)}^{2}}$$

(3) Adjusted coefficient of determination (*Adjusted R*^2^):

(10) $$R_{adjusted}^{2}=1-\frac{\left(1-R^{2}\right)\left(n-1\right)}{n-p-1}$$

(4) Root mean squared error (*RMSE*):

(11) $$RMSE=\sqrt{\frac{1}{n}\sum_{i=1}^{n}{\left({\hat{y}}_{i}-y_{i}\right)}^{2}}$$

where ${\hat{y}}_{i}$ is the predicted value, $y_{i}$ is the true value, ${\bar{y}}_{i}$ is the mean of the true value, *n* is the number of samples, and *p* is the number of features.

The results of the evaluation indexes for each function are summarized in Table 3. As shown in Table 3, the worst performance is exponential function with *SSE* of 8.48 × 10^−5^ and *RMSE* of 0.0092, both of which are the maximum among the three functions. In addition, the *R*^2^ and *Adjusted R*^2^ of the exponential function are the smallest, at 0.9837 and 0.9350 respectively. The linear rational number approximation function and the quadratic rational number approximation function have their own performance advantages. The *Adjusted R*^2^ and *RMSE* results of the linear rational number approximation function are superior to those of the quadratic rational approximation function, while the *SSE* and *R*^2^ perform slightly worse. On the whole, we consider that the values of *SSE*, *R*^2^ and *Adjusted R*^2^ are less different, and the effect of linear rational number approximation function is better in *RMSE*. Therefore, this paper adopts linear rational number approximation function as the final model, and its *SSE*, *R*^2^, *Adjusted R*^2^ and *RMSE* values are 5.63 × 10^−5^, 0.9892, 0.9784 and 0.0053, respectively.

**Figure 6:**
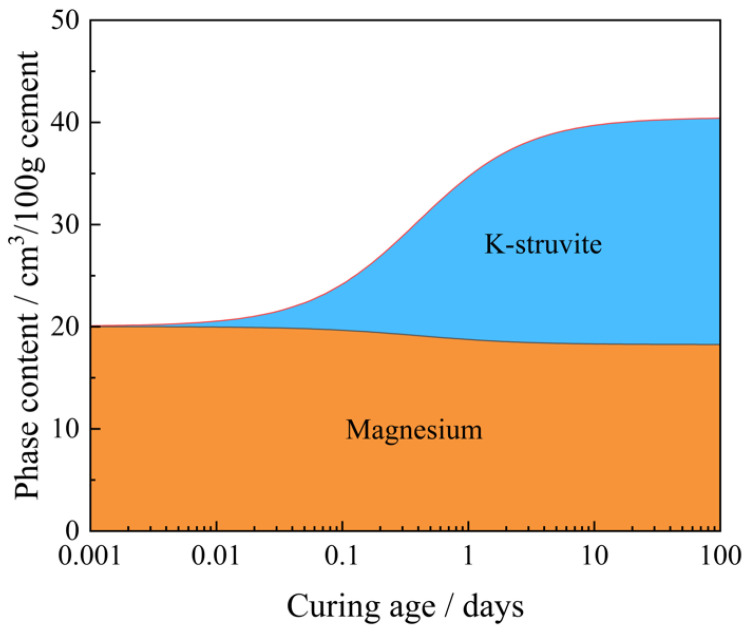
Changes in the content of MgO and K-struvite during hydration of MKPC.

The prediction of the changes in the hydration products of MKPC is completed using the thermodynamic data presented in Section 2.2.5, which is input into GEMS software. Figure 6 shows the changes in the content of MgO and K-struvite in the MKPC system at different curing ages by GEMS thermodynamic calculations. As shown in the figure, the hydration reaction of MKPC is fast, and a large amount of MgO is still present in the system at equilibrium in addition to the hydration product K-struvite, which is the same as the experimental results in this paper. The prediction results of GEMS show that when the contents of the reactants are varied and this variation is expressed as a function of time, based on accurate and complete thermodynamic data, GEMS can reliably calculate the contents of each substance for different curing ages at equilibrium under current system conditions. This is accomplished using its built-in thermodynamic logic. The application of thermodynamic modeling methods to the field of MKPC can effectively advance the study of MKPC hydration.

## 4. Conclusions

The hydration behavior of MKPC with an M/P molar ratio of 8.5 and a water-to-cement ratio of 0.22 was investigated through a series of experimental characterization methods and thermodynamic modeling. The conclusions are as follows:

(1) Dead burnt MgO reacts with KH_2_PO_4_, hardening to produce MKPC. K-struvite is the main hydration product, with a plate-like crystalline phase. The hydration rate of MKPC is very fast, and its hydration reaction process mainly occurs before 1 day.
(2) With the deepening of the degree of hydration, the content of the hydration product K-struvite increases, the pores in the system decrease, and the pore diameter distribution shifts toward the direction of small pores, with the structure becoming denser.
(3) Thermodynamic modeling effectively analyzes the content changes in each phase during the hydration of MKPC. The thermodynamic modeling methods have effectively promoted the study of the hydration of MKPC.

## Figures and Tables

**Table 1:** Chemical composition of the MgO (wt%).

Materials	MgO	SiO_2_	CaO	Al_2_O_3_	Fe_2_O_3_	SO_3_
Magnesia	85.1	7.9	3.3	2.4	0.9	0.1

**Table 2:** Thermodynamic data of each substance.

Species	Molecular Weight	log K° ^1^	Δ_f_G°	Δ_f_H°	S°	C_P_° ^2^	Vol ^3^
			[kJ/mol]	[kJ/mol]	[J/mol/K]	[J/mol/K]	[cm^3^/mol]
Brucite Mg(OH)_2_	58	16.84	−832.23	−923.27	63.14	77.28	24.6
Periclase MgO	40		−569.38	−601.66	26.95	37.8	11.3
K-struvite MgKPO_4_·6H_2_O	266	−10.96	−3240.8	−3717.3	350.1	324.8	142.5
KDP KH_2_PO_4_	136		−1415.9	−1568.3	134.9	116.6	58.2

^1^ K is the equilibrium constant. Solubility products refer to reactions formulated with H^+^, K^+^, Mg^2+^, PO_4_^3+^ and H_2_O. ^2^ C_P_° is the specific heat at constant pressure. ^3^ Vol is the molar volume. Normally, the data in the table refer to the reference temperature of 25 °C (298.15 K) and atmospheric pressure.

**Table 3:** Summary of evaluation indicators of function fitting.

Number	Function	*SSE*	*R* ^2^	*Adjusted R* ^2^	*RMSE*
1	Exponential	8.48 × 10^−5^	0.9837	0.9350	0.0092
2	Rational_linear	5.63 × 10^−5^	0.9892	0.9784	0.0053
3	Rational_quadratic	4.99 × 10^−5^	0.9904	0.9617	0.0071
